# Health and health-related indicators in slum, rural, and urban communities: a comparative analysis

**DOI:** 10.3402/gha.v9.33163

**Published:** 2016-12-02

**Authors:** Blessing U. Mberu, Tilahun Nigatu Haregu, Catherine Kyobutungi, Alex C. Ezeh

**Affiliations:** African Population and Health Research Center, Nairobi, Kenya

**Keywords:** slum, rural, urban health, health, comparative analysis

## Abstract

**Background:**

It is generally assumed that urban slum residents have worse health status when compared with other urban populations, but better health status than their rural counterparts. This belief/assumption is often because of their physical proximity and assumed better access to health care services in urban areas. However, a few recent studies have cast doubt on this belief. Whether slum dwellers are better off, similar to, or worse off as compared with rural and other urban populations remain poorly understood as indicators for slum dwellers are generally hidden in urban averages.

**Objective:**

The aim of this study was to compare health and health-related indicators among slum, rural, and other urban populations in four countries where specific efforts have been made to generate health indicators specific to slum populations.

**Design:**

We conducted a comparative analysis of health indicators among slums, non-slums, and all urban and rural populations as well as national averages in Bangladesh, Kenya, Egypt, and India. We triangulated data from demographic and health surveys, urban health surveys, and special cross-sectional slum surveys in these countries to assess differences in health indicators across the residential domains. We focused the comparisons on child health, maternal health, reproductive health, access to health services, and HIV/AIDS indicators. Within each country, we compared indicators for slums with non-slum, city/urban averages, rural, and national indicators. Between-country differences were also highlighted.

**Results:**

In all the countries, except India, slum children had much poorer health outcomes than children in all other residential domains, including those in rural areas. Childhood illnesses and malnutrition were higher among children living in slum communities compared to those living elsewhere. Although treatment seeking was better among slum children as compared with those in rural areas, this did not translate to better mortality outcomes. They bear a disproportionately much higher mortality burden than those living elsewhere. Slum communities had higher coverage of maternal health services than rural communities but it was not possible to compare maternal mortality rates across these residential domains. Compared to rural areas, slum communities had lower fertility and higher contraceptive use rates but these differences were reversed when slums were compared to other urban populations. Slum–rural differences in infant mortality were found to be larger in Bangladesh compared to Kenya.

**Conclusion:**

Mortality and morbidity indicators were worse in slums than elsewhere. However, indicators of access to care and health service coverage were found to be better in slums than in rural communities.

## Introduction

In the developing world, 881 million people were estimated to be living in slums in 2014 as compared to 792 million in 2000 and 689 million in 1990. The figure has been growing by about 9 million a year since 2000. The growth is not uniform across different regions. For instance, sub-Saharan Africa (SSA) is unique in that it is the only region where majority of its urban population live in slums. A recent UN-Habitat estimate puts this proportion at 56%. About 201 million people in SSA were living in slums in 2014. Although the proportions are declining, the absolute number of people living in slums continues to grow. Between 2000 and 2014, slum population in SSA increased by an additional 72 million people (1, 2).

The health of the slum population matters a lot for a number of reasons. First, it has high significance as nearly 1 billion people (and counting!) live in slums. Slums are not going away soon. Slum health will increasingly determine both urban and national health indicators. Second, the transition gradient is highly variable. Slums are no longer a half-way point on the route from rural poverty to urban middle class. For a large majority, slum is their home. Third, there are unfair differences in health. All data consistently show that health in slum populations is much worse than in other urban areas. Fourth, the visibility of this inequity, especially as developing country economies continue to grow, has implications for political stability and increased radicalization of the youth. Fifth, there is untapped potential for impact. Slums have high densities, more-educated, and youthful populations that can amplify the impact of public health interventions. Finally, the public health impacts of health problems in slum areas are immense. Natural and social barriers that support the management of outbreaks are porous in urban settings, and outbreaks are more difficult to control once they are in the urban space – indeed they quickly become global concerns; SARs and Ebola are examples (3–6).

It is generally expected (and unfortunately acceptable to many) that slum populations have poorer health status including morbidity, mortality, and health risks as compared with other urban populations (7). Part of this uncritical acceptance is the belief that they, slum residents, after all, are better off than rural residents. Besides, it is also acknowledged that all slums are not equal and variations in health status among slum population groups are as evident as variations across rural communities (8).

Although some people consider life in a slum as an opportunity to transition to middle class groups, others describe it as a poverty trap. Although satisfaction/dissatisfaction among slum residents could be affected by many economic, social, and environmental factors as well as the initial reasons for moving in to the slums, existing evidence suggests that most slum residents in low- and middle-income countries are not satisfied but have to stay in slums (9). Studies have also indicated that the descriptive evidence of consistent differentials in mortality and morbidity substantiates a view that urban poverty is a complex mix of material and social deprivation for developing countries and that both elements have intricate and possibly synergistic effects on individual and group health (10–12).

The perceived better access to health care among slum dwellers relative to rural residents further stultifies any sense of injustice slum residents may be suffering. However, recent data from Kenya and Bangladesh challenge the idea that slum dwellers enjoy better health than rural populations (13, 14). Some of these studies have been dismissed as not being the representative of slums, and the lack of appropriate data on slum-specific health indicators has frustrated efforts to clarify whether slum residence is health-enhancing or damaging to the health of the residents. Although cross-sectional data are limiting in answering these fundamental questions, the ability to compare health outcomes between slum and rural populations sheds light on potential advantages or disadvantages slum residents may have relative to a group that is generally accepted as being more vulnerable to poor health outcomes. The primary objective of this study, therefore, was to compare health and health-related indicators among slum, rural, and urban populations and how these are related to national and urban/city averages.

## Methods

### Research question

Do slum populations in low- and middle-income countries have better/worse/similar health and health-related indicators (in terms of risk, morbidity, access to care, and mortality) as compared with respective populations in other residential domains?

### Study design

We used a comparative analysis of health status indicators among slums, rural, and urban populations in four developing countries. For countries with two data points, differences between health status indicators at the two time points and rate of change in health indicators between the two points were also compared to highlight how slum and rural health indicators have changed over time. Across the four countries, within-country differences in health indicators and differences in average annual rates of change in the health indicators were also compared.

### Study countries

We purposively selected Bangladesh, Kenya, Egypt, and India for this study. This selection was informed by the availability of representative and comparable slum data for a specific city or for all urban areas in a country from household surveys and/or demographic and health surveys (DHS) that can be compared to other available data for rural, city, or urban areas in the same country over the same time period.

### Data sources

For Bangladesh, urban health surveys (encompassing city corporations and other urban areas) conducted in 2006 and 2013 were used as the main sources of data for slum health indicators (15, 16). For rural and urban health indicators, DHS conducted in 2007 and 2014 were the data sources (17, 18). Similarly, Nairobi Cross-sectional Slum Surveys (NCSS) conducted in 2000 and 2012 were the sources of data on slum health indicators for Nairobi, Kenya (13, 19). Kenya DHS 2003 and 2014 were the sources of data for rural and urban health indicators in Kenya (20, 21). For Egypt and India, indicator data for all residential domains were extracted from 2003 and 2005 to 2006 DHS reports, respectively (22–25).

### Identification of slums in the four countries

#### Bangladesh

The 2006 Urban Health Survey was conducted in six megacities in Bangladesh. Slums were defined as areas of concentrated vulnerability. Using satellite images from census 2005 as a starting point, four criteria for identifying slums were used: poor housing conditions, high overall density, poor environmental services, and high prevalence (over 75%) of people with income below the poverty level. If an urban area was comprised of at least 10 households or was a mess unit with at least 25 members and appeared to satisfy these criteria, it was entered to complete a checklist of the key characteristics of the settlement. In the 2013 survey, nine city corporations were included. The same criteria for identification of slums were used in both surveys.

#### Egypt

The focus of the interim 2003 DHS data was on greater Cairo slums. These included urban populations living in the slum areas of Cairo, Giza, and Kalyubia governorates. A list of slums compiled by the Central Agency for Public Mobilization and Statistics (CAPMAS) was used as a sampling frame. CAPMAS used elements, such as high population density, substandard housing, and social disorganization, to identify and list slums. An area was included in the list if it was unplanned, it lacked basic services, the majority of buildings were constructed without permits, streets were unstructured, and the population was poor and uneducated.

#### India

The identification of slums was based on the 2001 Census. In the 2001 Census, the following criteria were used to designate the area as slum or non-slum: ‘1) all specified areas in a town or city notified as “Slum” by State/Local Government and UT Administration under any Act including a “Slum Act”; 2) all areas recognized as “Slum” by State/Local Government and UT Administration, Housing and Slum Boards, which may have not been formally notified as slum under any act; and 3) a compact area of at least 300 population or about 60–70 households of poorly built congested tenements, in unhygienic environment usually with inadequate infrastructure and lacking in proper sanitary and drinking water facilities’. In India, slums are declared legally and are to be notified by a competent administrative authority. The objective of declaring an area as a slum is basically to be able to allocate funding to extend or improve upon civic services. All notified slums are considered to be legal slums, which tend to be of a permanent nature. The municipal body is expected to provide all civic services to such areas. Census slums in Mumbai were the focus of this analysis.

#### Kenya

The selection of slums for the survey was informed by the 1999 Census. Based on census enumeration areas used in the 1999 Kenya National Census, a weighted cross-sectional sample was designed that is representative of households in all slum clusters in eight administrative locations of Nairobi. Both the 2000 and 2012 NCSS were conducted by the African Population and Health Research Center.

The DHS surveys, the sources of data for rural population for this study, didn’t define urban–rural in the selected countries. They have adopted the country’s rural definition. So the urban–rural definition is not addressed in a DHS report. The definition is country-specific and may vary from country to country. In some countries, it is based on population density, and in others, it is based on infrastructure.

### Health status indicators

Based on availability and comparability of indicators across the selected countries, data for the following health indicators were extracted:

#### Child health indicators

Neonatal mortality, post-neonatal mortality, infant mortality, child mortality, under-five mortality, child nutrition, immunization, breastfeeding, as well as prevalence and treatment of acute respiratory infections (ARI), fever, and diarrhea.

#### Maternal health indicators

Antenatal care (ANC) coverage, place of delivery, skilled birth attendance, and postnatal care for the mother and the newborn.

#### Reproductive health indicators

Age at first marriage, age at first sexual intercourse, age at first birth, contraceptive prevalence rate, unmet need for family planning, total fertility rate, and teenage pregnancy and motherhood rates.

#### HIV/AIDS indicators

Knowledge of the disease, its transmission and prevention, perception toward people with the disease, and HIV testing status.

### Data extraction

Indicator data for the different population groups in the four selected countries were extracted from the respective survey reports into a matrix, and relevant details associated with the measurement and computation of these indicators were also noted. The selection of standard indicators from the DHS enabled direct comparison of the indicator values and direct computation of the differences.

### Analysis and synthesis

#### Within-country comparison

We compared health indicators for slum, rural, and urban populations at two time points for Kenya and Bangladesh. In Bangladesh, slum health indicator data from Urban Health Survey 2006 were compared with rural health indicators from DHS 2007 and those from Urban Health Survey 2013 data with DHS 2014 data. In Kenya, slum health indicators from the NCSS 2000 were compared with Kenya Demographic and Health Survey (KDHS) 2003 data and those from NCSS 2012 with KDHS 2014 data. Differences in the indicator values between slum and rural populations were computed. For India and Egypt, indicators from the same DHS surveys were compared.

#### Within-country comparison (between two time points)

In Bangladesh, the changes in slum health indicators between 2006 and 2013 were compared with the changes in rural health indicators between 2007 and 2014. Similarly, in Kenya, the changes in slum health indicators between 2000 and 2012 were compared with the changes in rural health indicators between 2003 and 2014. India and Egypt had data at a single point, and this comparison was not possible.

#### Between-country comparison

The differences between the slum–rural differences (differences in differences) were compared between the two countries for the period 2012–2014 Average annual changes (AACs) were computed by dividing the total change in an indicator between two time points by the number of years between the two time points. The differences in the AACs in slum and rural health indicators were also compared between the countries. Findings were presented using numerical summaries and tables.

## Findings

### Bangladesh

According to UN-Habitat report, about 55% of Bangladesh’s urban population, an equivalent of about 29 million people, lived in slum areas in 2014. Urban slums deserve special attention in Bangladesh as they host a significant proportion of the total population of the country (1, 2). Although the proportion of the urban population in Bangladesh living in slums has declined from 87% in 1990 to 55% in 2014, the absolute number of slum dwellers in the country has increased by almost 50% (by about 10 million people) between the two time points.

#### Child health indicators

Slums had higher early childhood mortality as compared to rural, urban, and non-slum populations in Bangladesh. Comparison of 2006–2007 childhood mortality indicators in Bangladesh showed that slum populations had higher mortality rates except for the child mortality rate, which was similar between slum and rural population groups. In the years 2006–2007, both infant mortality rate and under-five mortality rate were higher in Bangladesh slums than in non-slums, urban, and even rural areas. Infant mortality rate in slums was more than two times higher than in non-slum areas. Slums in Dhaka had about 2.6 times higher under-five mortality rates than the corresponding non-slum areas. It was also demonstrated that under-five mortality in slums was higher (95 per 1,000) than in rural (66 per 1,000). The 2013–2014 indicator data had also a similar pattern apart from the fact that neonatal mortality rate was the same in both population groups (Table 1).

**Table 1:** Health status indicators in Bangladesh for slums (S), non-slums (NS), rural (R), urban (U), Dhaka (DK), and national (N) populations

	UHS 2006		BDHS 2007				UHS 2013		BDHS 2014			
				
Childhood mortality	Slum	Non-slum	Rural	Urban	Dhaka	National	Slum	Non-slum	Rural	Urban	Dhaka	National
Neonatal mortality rate	43.7	20.1	41.0	33.0	38.0	37.0	31.0	NA	31.0	21.0	25.0	28.0
Post-neonatal mortality rate	19.3	9.6	18.0	17.0	18.0	15.0	18.0	NA	9.0	13.0	10.0	10.0
Infant mortality rate	63.1	29.8	59.0	50.0	55.0	52.0	49.0	NA	40.0	34.0	35.0	38.0
Child mortality rate	18.8	1.3	19.0	13.0	14.0	14.0	9.0	NA	10.0	3.0	5.0	8.0
Under-five mortality rate	80.7	31.0	77.0	63.0	69.0	65.0	57.0	NA	49.0	37.0	41.0	46.0
Child health												
Stunting (%)	55.9	36.0	45	36.4	44.0	43.2	49.6	33.4	37.9	30.8	33.9	36.1
Wasting (%)	17.4	9.8	18.2	14.4	15.4	17.4	18.5	16.4	15.1	12.2	11.9	14.3
Underweight (%)	45.6	28.1	43	33.4	39.9	41.0	42.5	26.4	34.8	26.1	28.5	32.6
ARI prevalence (%)	14.2	12.2	5.2	3.3	11.6	13.0	2.6	2.3	5.7	4.3	5.2	5.4
ARI treatment (%)	40.6	72.6	33.8	56.6	31.7	30.2	56.2	66.1	39.3	52.1	43.2	42
EBF (median, months)	2.4	1.5	1.8	1.6	1.0	1.8	3.1	2.8	2.8	2.7	NA	4.0
PBF (median, months)	3.7	3.1	3.2	3.1	2.0	3.2	3.4	2.8	4.3	4.6	3.1	5.6
Maternal health												
Antenatal care	62.3	84.7	46.4	71.3	48.2	51.7	53.8	83.2	58.6	78.8	64.3	63.9
Skilled birth attendance	18.0	56.0	13.2	36.7	19.8	18.0	37.3	67.7	35.6	60.5	43.5	42.1
Health facility delivery	12.3	46.3	10.5	30.6	16.9	14.6	36.7	65.1	30.6	56.8	40.5	37.4
Postnatal care (mother, 2 days)	13.7	40.6	16.5	39.0	19.8	18.5	34.0	60.4	29.5	55.9	36.8	36.4
Postnatal care (child, 2 days)	13.3	42.1	17.0	40.0	19.8	18.5	26.5	49.2	24.6	50.9	30.5	31.5
Reproductive health												
Total fertility rate	2.5	1.8	2.8	2.4	2.8	2.7	2.0	1.7	2.4	2.0	2.3	2.3
Current use of contraception	53.2	55.6	46.0	52.4	47.5	47.5	62.3	56.4	53.2	56.2	54.2	54.1
Birth interval	45.0	52.0	42.8	46.8	44.0	43.6	54.0	60.0	50.5	55.5	53.9	51.7
Median age at first birth	17.4	18.7	17.7	18.7	18.2	18.2	18.2	19.4	18.1	18.4	18.5	18.4
Teenage pregnancy (first) (%)	4.4	8.3	6.0	6.2	6.4	6.1	4.8	3.2	6.1	6.4	6.2	6.2
Teenage motherhood (%)	17.0	2.2	29.2	18.1	27.0	26.6	16.6	10.1	26.0	21.0	25.6	24.6

ARI, acute respiratory infections; EBF, exclusive breastfeeding; PBF, predominant breastfeeding; NA, indicator data not available.

Under-five children’s nutritional status in slums was relatively lower than that of rural, non-slum, and urban children at both points of comparison. The difference is remarkable in the prevalence of stunting. The prevalence of stunting among rural children in Bangladesh is lower (as compared with slums) by more than 10 percentage points at both time points. The difference in prevalence of underweight was more remarkable in 2013–2014.

**Table 2:** Health indicators in Kenya for slums, rural, urban, Nairobi, and national populations

		KDHS 2003					KDHS 2014			
				
Childhood mortality^a^	NCSS 2000 Slum	Rural	Urban	Nairobi	National	NCSS 2012 Slum	Rural	Urban	Nairobi	National
Neonatal mortality rate	30.4	34.0	26.0	32.0	33.0	14.4	21.0	26.0	39.0	22.0
Post-neonatal mortality rate	60.9	44.0	36.0	35.0	44.0	24.9	18.0	16.0	16.0	16.0
Infant mortality rate	91.0	79.0	61.0	67.0	77.0	39.2	40.0	43.0	55.0	39.0
Child mortality rate	65.2	41.0	35.0	30.0	41.0	40.6	16.0	15.0	17.0	14.0
Under-five mortality rate	151.0	117.0	93.0	95.0	115.0	79.8	56.0	57.0	22.0	52.0
Child health										
Vaccinated by 12 months (all)	41.3	56.4	58.7	63.1	56.8	45.2	77.4	83.0	81.2	79.4
Prevalence of fever	67.9	40.8	39.8	38.5	40.6	17.2	25.9	21.7	18.7	24.4
Treatment of fever	63.5	43.6	53.6	56.3	45.5	65.0	62.6	62.3	63.3	62.5
Prevalence of ARI/cough	43.8	18.9	16.4	16.4	18.4	24.6	9.1	7.3	5.9	8.5
Sought treatment of ARI/cough	66.3	43.6	53.6	56.3	45.5	66.6	66.7	63.6	65.2	65.7
Prevalence of diarrhea (all types)	30.8	15.8	17.0	13.9	16.0	20.2	15.7	14.3	15.6	15.2
Treatment of diarrhea	57.8	29.5	30.7	35.0	29.7	42.7	58.1	56.7	57.4	57.6
Maternal health										
Antenatal care	96.0	86.8	93.2	95.4	88.1	96.2	94.0	97.8	97.6	95.5
Tetanus vaccination (TT2 +)	61.0	50.6	57.2	55.3	51.9	62.3	47.6	56.5	60.3	51.1
Skilled birth attendance	52.3	34.5	72.0	79.0	41.6	82.6	50.4	82.4	89.1	61.8
Health facility delivery	54.3	33.2	70.2	77.9	40.1	83.0	49.5	82.0	88.7	61.2
Reproductive health										
Total fertility rate	4.0	5.4	3.3	2.7	4.9	3.5	4.5	3.1	2.7	3.9
Median age at 1st marriage	20.2	19.3	21.4	22.1	19.7	22.0	19.5	21.5	22.1	20.2
Median age at 1st sex (25–49)	16.3	17.4	18.6	19.2	17.8	19.0	17.3	18.8	17.8	17.4
Birth interval (median, months)	34.1	32.1	36.0	34.9	32.6	36.3	34.7	41.0	43.7	36.3
Median age at first birth (20–49)	19.9	19.4	21.2	22.0	19.8	20.0	19.7	21.3	22.2	20.3
Current use of contraception	39.0	29.2	39.9	44.3	31.5	40.1	50.9	56.9	58.3	53.2
Total demand for family planning	66.5	65.8	66.1	68.1	65.8	78.8	75.2	75.9	73.4	75.5
Unmet need for family planning	23.3	26.6	17.2	16.0	24.5	23.7	20.2	13.4	11.1	17.5
HIV/AIDS										
HIV/AIDS awareness	93.4	98.3	99.2	99.6	98.5	90.3	99.6	99.8	99.8	99.7
Knowledge – abstinence	35.7	77.8	83.5	86.2	79.2	47.8	83.2	83.9	82.8	83.5
Knowledge – being faithful	31.0	78.9	85.2	88.7	80.5	21.2	90.4	93.3	94.7	91.6
Knowledge – condom use	56.4	58.3	69.2	75.2	61.0	71.7	77.1	83.7	86.8	79.8
Correct perception of HIV status	89.7	88.7	93.6	90.9	84.7	92.2	86.2	91.8	92.2	88.5
Knowledge of vertical trans.	91.8	70.2	76.5	77.4	71.8	88.8	87.8	89.7	91.4	88.5
Use condom at higher-risk sex	25.4	19.5	33.0	31.7	23.9	32.0	29.6	58.7	45.7	42.8
Ever tested for HIV (women)	27.1	13.4	24.7	28.8	16.2	93.5	82.2	88.7	90.9	84.9

a Rural, urban, and Nairobi mortality rates are for the 10 years preceding the survey while national estimates is for 5-year period. NCSS is also 5-year period.

**Table 3:** Health indicators in Egypt for slum, all urban areas, urban governorates, rural, and national populations.

	Egypt interim DHS 2003				
	
Childhood mortality	Slum	All urban	Urban gov.	Rural	National
Neonatal mortality rate	21.8	17.6	NA	NA	22.7
Post-neonatal mortality rate	10.0	5.5	NA	NA	15.1
Infant mortality rate	31.8	23.1	26.3	51.4	38.0
Child mortality rate	5.9	8.3	NA	NA	7.9
Under-five mortality rate	37.5	31.2	33.5	63.1	45.7
Child health					
Stunting	14.8	15.2	15.6	16.6	15.6
Wasting	0.9	1.0	3.1	4.1	4.0
Underweight	6.3	7.2	5.7	9.6	8.6
Vaccinated by 12 months (fully)	93.0	87.6	87.2	86.7	87.5
Prevalence of ARI/cough	14.7	11.3	12.5	9.7	10.2
Sought treatment of ARI/cough	75.4	63.9	69.8	71.6	70.2
Prevalence of diarrhea (all diarrhea)	23.9	18.6	17.8	20.2	18.9
Treatment of diarrhea	47.2	43.1	39.8	45.5	45.7
EBF (median, months)	0.7	2.0	1.5	1.6	1.5
Full breastfeeding (median, months)	1.6	2.5	2.0	2.6	2.4
Maternal health					
Antenatal care	77.1	80.9	83.9	60.3	68.7
Tetanus vaccination (TT1+)	70.4	63.4	75.4	44.9	43.4
Skilled birth attendance	84.0	89.7	90.2	59.0	69.4
Health facility delivery	79.3	82.6	82.5	47.7	59.0
Postnatal care (mother, 2 days)	47.1	49.1	42.3	23.6	29.1
Postnatal care (child, 2 days)	36.6	45.2	44.5	16.1	23.5
Reproductive health					
Total fertility rate	3.1	2.3	2.2	3.6	3.2
Median age at first marriage	20.6	21.5	NA	19.1	20.0
Birth interval (Median # of months)	38.2	40	39.8	34.1	35.5
Median age at first birth (20–49 years)	22.3	23.5	24.0	20.8	22.1
Contraceptive prevalence rate (%)	61.6	64.0	64.5	53.0	56.6
Total demand for family planning (%)	72.8	73.2	74.3	69.0	68.2
Unmet need for family planning (%)	7.1	5.0	5.1	12.0	11.2
Teenage pregnancy (first)	1.9	1.0	1.0	3.0	2.5
Teenage motherhood	3.6	2.6	2.3	7.5	5.6
HIV, HCV, and FGC					
Knowledge of HIV/AIDS (awareness)	97.9	98.6	97.9	83.9	89.6
Knowledge of HIV transmission (1 way)	83.5	86.9	86.2	67.2	76.2
Knowledge of HCV (awareness)	75.1	84.7	86.0	53.1	65.2
Knowledge of HCV transmission	53.4	60.3	62.8	46.7	55.9
Married women circumcised	98.1	95.8	91.3	98.8	97.0
Women who support female circumcision	69.7	57.8	50.5	81.8	71.1
Women who want their daughter circumcised	30.6	24.3	19.3	34.8	37.6

ARI, acute respiratory infections; HCV, hepatitis C virus; NA: indicator values were not computed in the respective data sources.

**Table 4:** Health indicators in India for Mumbai slums, Mumbai non-slums, Mumbai, all urban areas, rural, and national populations

	IDHS 2005–06					
	
Childhood mortality	Slum	Non-slum	Mumbai	Urban	Rural	National
Infant mortality rate	24.9	40.1	30.3	41.5	62.2	57
Under-five mortality rate	32.7	43.6	36.6	51.7	82.0	74.3
Child health						
Stunting (<–2SD)	47.4	41.5	45.4	39.6	50.7	48.0
Wasting (<–2SD)	16.1	16.4	16.2	16.9	20.7	19.8
Underweight (<–2SD)	36.1	25.8	32.6	32.7	45.6	42.5
Vaccinated by 12 months (fully)	68.7	72.5	69.8	57.6	38.6	36.3
Prevalence of ARI/cough	1.6	1.7	1.7	5.1	6.0	5.8
Prevalence of diarrhea (all diarrhea)	6.8	4.7	6.1	8.9	9.0	9.0
Prevalence of fever	9.8	6.0	8.5	14.0	15.1	14.9
Children age 0–71 months covered by AWC service	48.6	19.2	25.2	NA	NA	81.1
Children 0–71 months received any AWC service	15.7	6.3	14.0	23.4	34.8	32.9
Prevalence of anemia in children (any)	50.2	46.9	49.1	63.0	71.5	69.5
Maternal health						
Antenatal care (3+)	90.3	93.0	91.3	89.1	67.5	74.2
Tetanus vaccination (TT2+)	89.7	91.5	90.3	86.4	72.6	76.3
Received all recommended ANC care	18.3	22.1	19.7	NA	NA	15.0
Skilled birth attendance	92.5	82.2	85.7	73.5	37.5	46.6
Health facility delivery	83.3	91.2	86.0	67.5	28.9	38.7
Postnatal care ( within 2 days)	62.3	76.9	67.5	61.0	28.6	37.3
Reproductive health						
Total fertility rate	1.9	1.4	1.68	2.06	3.0	2.7
Contraceptive prevalence rate (%)	51.4	61.1	55.5	55.8	45.3	56.3
Teenage pregnancy (first)	2.7	0.0	1.5	2.4	4.6	3.9
Teenage motherhood	7.1	2.9	5.2	6.3	14.5	12.1
Physical or sexual violence ever (women)	22. 9	14. 7	19. 3	28.3	36.1	33.5
HIV, TB, health care						
Knowledge of HIV/AIDS (awareness)	92.5	95.6	93.9	83.2	50.0	57
Knowledge of HIV/AIDS (comprehensive)	40.0	54.8	46.5	30.3	11.0	17.3
Heard of TB (women)	94.9	96.5	95.6	92.2	81.9	85.3
Source of health care – public medical sector	25.4	20.9	23.4	29.6	36.8	34.4
Other diseases and conditions						
Obesity (women) (BMI>30)	7.7	8.7	8.1	6.1	1.3	2.8
Underweight (women) (BMI<18.5)	23.1	21.4	22.4	25	40.6	35.6
Anemia in women (any)	46.0	47.9	46.8	50.9	57.4	56.2
No. of females per 100,000 with medically treated TB	810	482	667	246	337	309
No. of women per 100,000 who have diabetes	1,174	1,236	1,201	1,374	641	881
No. of women per 100,000 who have asthma	1,897	1,331	1,648	1,648	1,719	1,696
No. of women per 100,000 who have thyroid disorders	542	856	680	1,339	758	949
Percentage of men who use any kind of tobacco	45.9	34.6	41.3	28.7	35	32.7
Percentage of men who drink alcohol	36.2	28.7	33. 1	30.9	32.5	32.0

ARI, acute respiratory infections; ANC, antenatal care; AWC, anganwadi centre.

**Table 5:** Slum–rural differences (as percentage of rural) 2012–2014

	Slum–rural difference (%) Bangladesh	Slum–rural difference (%) Kenya
Neonatal mortality rate	0.0	−31.4
Post-neonatal mortality rate	100.0	38.3
Infant mortality rate	22.5	−2.0
Child mortality rate	−10.0	153.8
Under-five mortality rate	16.3	42.5

**Table 6:** Average annual changes (AACs) and slum–rural differences in AACs

	Bangladesh			Kenya		
		
	Slum	Rural	Diff (%)	Slum	Rural	Diff (%)
Child health						
Neonatal mortality rate	1.8	1.4	27.00	1.3	1.2	12.82
Post-neonatal mortality rate	0.2	1.3	−85.56	3.0	2.4	26.92
Infant mortality rate	2.0	2.7	−25.79	4.3	3.5	21.75
Child mortality rate	1.4	1.3	8.89	2.1	2.3	−9.80
Under-five mortality rate	3.4	4.0	−15.36	5.9	5.5	6.99
Maternal health						
Antenatal care	1.2	−1.0	−226.87	0.0	−0.6	−97.22
Skilled birth attendance	−2.8	−3.2	−13.84	−2.5	−1.4	76.16
Health facility delivery	−3.5	−2.9	21.39	−2.4	−1.3	91.33

Note: Difference (%)=[(slum−rural)/rural]*100.

However, the median duration of exclusive breastfeeding (EBF) was higher in slums than rural, non-slum, and urban areas at both time points. Median duration of predominant breastfeeding had decreased in slums but increased in rural areas. On the contrary, prevalence of ARI in slum areas had significantly decreased between the two time points while it stabilized at a low level in rural areas. More children with ARI in slum areas sought treatment for than in rural areas.

#### Maternal health services

Overall, slum areas generally had relatively higher coverage of maternal health services than rural population but a lower coverage as compared with non-slum and urban populations. However, a study in 2009 had shown that skilled birth attendance in slums (15%) was lower than in rural (19%). Contrary to the expectation, ANC coverage had decreased between 2006 and 2013 in slums although it increased in rural and urban Bangladesh between 2007 and 2014. On the contrary, postnatal care coverage had improved in all settings, but with a higher rate in the slums. Apart from these differences, slum and rural areas in Bangladesh had a low coverage of most of the maternal health services.

#### Reproductive health indicators

Rural populations in Bangladesh had higher total fertility rates, lower contraceptive prevalence rates, lower birth intervals, and higher prevalence of teenage pregnancy and motherhood as compared with slum populations. Although teenage motherhood is lower in slums than in rural and urban populations, an increase in prevalence of teenage pregnancy in slum areas was also observed.

In the earlier surveys, child mortality indicators for slums were not significantly different from those of rural populations in Bangladesh. However, children in slums have significantly higher levels of stunting and ARI compared to any other subpopulation including children in rural areas. However, slum women are likely to receive ANC, more likely to use contraceptives, and teenage pregnancy is significantly lower compared to rural areas. In the more recent surveys, however, although child health indicators have improved significantly in rural areas, they have remained stagnant or worsened over the years in slum populations in Bangladesh. Consequently, in the 2013–2014 surveys, slum children experienced worse health outcomes relative to rural children and children in other residential domains. Across all indicators, slum populations fare worse than non-slum, urban, and national averages.

### Kenya

Kenya is a lower middle-income country with a total population of nearly 45 million and 46% poverty headcount ratio at national level. In 2014, life expectancy in the country was about 62 years. A quarter of the total population of Kenya lives in urban areas. Kenya is home to one of the largest slums in the world, the *Kibera* slum. Nairobi has more than 40 areas designated as slums and about 56% of the country’s urban population lived in slums in 2014.

#### Child health indicators

In the earlier surveys in Kenya, early childhood mortality indicators, with the exception of neonatal mortality rate, were higher in slums than in rural areas. All child morbidity and health service indicators were also worse for slum children in Nairobi than those in rural areas of Kenya. Across the other residential domains, slum children fared much worse than children elsewhere in Kenya. In more recent surveys, child health indicators improved substantially in Kenya, but the disadvantage of slum children still persisted. They are significantly more likely to have diarrhea and ARI/cough, less likely to get treatment for diarrhea, and less likely to be vaccinated (Table 2). The child mortality rate is more than double for slum children compared to those in any other residential domain in Kenya.

In the years 2012–2013, the under-five mortality rate in Nairobi slums was higher than all other estimates for urban, rural, Nairobi, and national levels. Although infant mortality was not remarkably higher in slums, the under-five mortality in Nairobi slums was about 3.6 times higher than that of Nairobi as a whole. The difference in infant mortality rate between slum and rural populations had narrowed down. However, there are still remarkable differences between slum, urban and rural populations in child mortality and under-five mortality rates. Overall, the decline in early childhood mortality was higher in slum areas than rural ones. Nairobi slums had remarkably lower coverage of immunization as compared with rural Kenya at both time points. The prevalence of childhood illnesses was also higher in slums than rural and urban areas. Treatment seeking for childhood illnesses was better in slums and urban than rural areas. The decline in the prevalence of childhood illnesses was faster in slums, whereas the increase in immunization coverage and treatment seeking behavior was higher in rural Kenya.

#### Maternal health service indicators

Slums in Kenya had a relatively higher coverage of ANC, skilled birth attendance, and institutional delivery than rural areas. Maternal health indicators had improved in both settings between the two time points. Although the improvement in ANC was higher for rural areas, the improvements in skilled birth attendance and institutional delivery in slums were almost two times the improvements in rural areas.

#### Reproductive health indicators

At the 2000–2003 time point, slum areas had lower total fertility rates and higher contraceptive prevalence rates as compared with rural Kenya. However, contraceptive prevalence rates in rural areas had increased by about twofold and had reversed the trend. The decline in total fertility rate was also about two times that of slum areas. Median ages at first marriage, first sexual intercourse, and first birth had increased in slums, but were more or less constant in rural areas across the two time points.

#### HIV/AIDS indicators

Awareness about HIV/AIDS was high in all three settings at both time points. Despite the improvements in both settings, knowledge about the three HIV prevention methods was lower in the slums than the rural areas at both time points. Slum areas had higher levels of HIV testing and condom use at both time points. Correct perception of one’s HIV status was similarly high in both settings at both time points.

### Egypt

#### Child health

In Egypt, in 2003, infant mortality rate was higher in slums as compared with all urban areas (32 vs. 23 per 1,000). Similarly, under-five mortality rate was also significantly higher in slums as compared with all urban areas in Egypt (37.5 vs. 31 per 1,000). However, both infant mortality rate and under-five mortality rate were lower than rural and national figures. The prevalence of ARI is about 1.3 times higher in slums as compared with all urban areas. Slum areas also had a 1.28 times higher prevalence of diarrhea in children. Median duration of EBF was about three times higher in urban areas than slums. Similarly, median duration of full breastfeeding was about 1.6 times higher in urban areas as compared with slums. Even rural areas had lower prevalence of childhood illnesses and higher duration of breastfeeding as compared with slums in Cairo (Table 3).

#### Maternal health

The coverage of key maternal health services didn’t significantly vary between slums and urban areas although urban areas had slightly higher coverages in most of the maternal health services. However, compared with rural areas, Cairo slums had much higher coverage of all maternal health service indicators included in this analysis. For instance, coverage of postnatal care for children within 2 days of delivery was about 2.3 times higher in Cairo slums as compared with rural areas in Egypt. Likewise, postnatal care for mothers within 2 days of delivery was about two times higher in Cairo slums as compared with rural average.

#### Reproductive health indicators

Cairo slums had a higher total fertility rate, lower median age at first marriage and first birth, a lower birth interval, a lower contraception use rate, and a higher unmet need for family planning as compared with all urban areas in Egypt. Teenage pregnancy was two times higher in slums than in urban areas. Teenage motherhood was also about 1.4 times higher in slums than in urban areas. However, rural areas had even higher levels of teenage pregnancy and teenage motherhood. Overall, reproductive health status was higher in urban areas than slums but worse in rural areas than in slums.

#### HIV, HCV and female circumcision

Awareness about HIV and its transmission was consistently high in all groups. Awareness about hepatitis C virus (HCV) infection and its transmission was low in rural areas as compared with slums and all urban areas. More women in slum areas than in urban areas support female circumcision and want to have their daughters circumcised. These figures are even higher in rural areas. In general, awareness-related indicators are better in Cairo slums than in rural areas.

### India

#### Child health

Unlike the other three countries, In Mumbai, during the years 2005–2006, the situation of child mortality in the census slums was different. Census slums in Mumbai had a lower infant mortality rate than census non-slums (25 vs. 40 per 1,000). Similarly, census slums had lower under-five mortality than census non-slums (33 vs. 44 per 1,000). These child mortality rates in Mumbai slums were even lower than the estimates for Mumbai urban averages, rural averages, and national figures. Childhood malnutrition was moderately higher in slums than in non-slums. But rural areas had much higher levels of childhood malnutrition in India. Prevalence of fever, diarrhea, and anemia were higher in slums than in non-slums. But rural areas had even much higher prevalence of these childhood health problems (Table 4).

#### Maternal health

Reception of all recommended ANC care was 21% higher in non-slum areas of Mumbai as compared with slums. Although skilled birth attendance was 11% higher in slums, health facility delivery was 9% lower in slums as compared with non-slum areas. Postnatal care coverage was about 23% higher in non-slums as compared with slums. Rural areas had lower coverages of all maternal health services as compared with slums.

#### Reproductive health and HIV/AIDS

As in the other three countries, total fertility rate, teenage pregnancy, and teenage motherhood were higher in slums and highest in rural areas. Use of modern contraception was higher in non-slums, lower in slums, and lowest in rural areas. There were no remarkable differences in awareness of HIV and TB among the comparison groups. However, comprehensive knowledge about HIV was lower in slums than in non-slums and much lower in rural areas. Experience of physical or sexual violence among women was 55% higher in slums than non-slums and 57% higher in rural areas than in slums.

#### Other diseases and conditions

Prevalence of obesity and underweight in women didn’t vary remarkably between slums and non-slums. However, the prevalence of obesity in women was about six times higher in Mumbai slums than in rural India. In contrast, the prevalence of underweight in women was almost two times higher in rural India than in Mumbai slums. Although diseases associated with micronutrient deficiency, anemia, and thyroid disorder were higher in rural areas than in slums, the prevalence of chronic diseases such as TB, diabetes, and asthma was significantly higher in slums. Use of tobacco and alcohol was higher in slums than in all other groups.

### Between-country comparison

Comparison of slum–rural differences in early childhood mortality indicators at the 2012–2014 study period showed that neonatal mortality in Kenyan slums was better than in rural Kenya, whereas it was same in Bangladeshi slums and rural Bangladesh. Post-neonatal mortality rate in Bangladeshi slums was double than that of rural Bangladesh, whereas in Kenyan slums it was 38% higher than rural Kenya. The difference in infant mortality between Bangladeshi slums and rural Bangladeshi was minimal. But this difference was 22.5% in Kenya illustrating that Kenyan slums are at a higher disadvantage than rural Kenya. The slum–rural difference in child mortality rate in Kenya was the highest (154%) indicating that children in slums were about 1.5 times more likely to die as compared with rural children. Slum–rural difference in under-five mortality was 16% in Bangladesh and 43% in Kenya (Table 5).

Slum–rural difference in ANC was –8% in Bangladesh and +2% in Kenya. The same difference in skilled birth attendance was 5% in Bangladesh and 64% in Kenya. For health facility delivery, this difference was 20% in Bangladesh and 68% in Kenya. This shows that slums in Kenya were better in maternal health services than slums in Bangladesh when both are compared to their rural counterparts.

Women in Bangladeshi slums gave birth to an average of 0.4 less children as compared with those in rural Bangladesh, whereas women in Kenyan slums gave birth to on average one child less than those in rural Kenya. Slum–rural difference in coverage of modern contraceptives was 9 and −11 percentage points in Bangladesh and Kenya, respectively. Differences in birth interval were 3.5 and 1.6 months in Bangladesh and Kenya, respectively. Age at first birth was higher in Kenya than Bangladesh with minimal slum–rural differences.

Slum–rural differences in AACs were positive for neonatal mortality rate and child mortality rate in Bangladesh and negative for child mortality rate in Kenya. Slum–rural difference in AACs was positive for health facility delivery in Bangladesh and negative for ANC in Kenya (Table 6).

## Discussion

This study compared large-scale multicountry data to describe health and health-related indicators in slums relative to other population groups. In this analysis, we have found higher levels of childhood mortality in slums than in non-slums and even than in rural areas. The only exception to this was slums in Mumbai which had lower levels of infant and under-five mortality rates. This exception could be because most of early childhood deaths occur in the neonatal period as documented in another study in an Indian slum (26). The definition of slums in India was also different from other countries.

Childhood morbidity, as illustrated by prevalence of ARI, diarrhea, fever, and anemia in the different countries, was found to be higher in slums than in all other groups of populations. Other studies have also found a high burden of childhood illnesses and childhood malnutrition in slum populations (27, 28). These illnesses could be due to the appalling inequalities in the distribution and access to basic amenities and health services between slum, non-slum, and rural populations (29). Access to treatment services and treatment seeking behavior were low in slums. Added up on higher levels of childhood morbidity, the consequences of these would be grave for the health status of children in slums.

The coverage of most of maternal health services was better in slums than in rural areas. This could be because access to health facilities was within their reach in the slum areas. But the coverage of maternal health services in slums was generally much lower than urban average. Although geographic access to maternal health services could be high in slums, financial access and quality of services may not be comparable to other settings. Besides, slums are not equal and the possibility of inequalities of maternal health service coverages within slums is usually high (30). Moreover, a study in Nairobi has reported that despite the high prevalence of ANC, the proportion of women who made the recommended number of visits or who initiated the visit in the first trimester of pregnancy remains low compared to Nairobi as a whole and, more importantly, compared to rural populations (31).

Slums generally have a higher fertility rate and a lower contraceptive prevalence rate as compared with other urban areas. However, in rural areas, fertility rates are much higher, and contraceptive prevalence rates are much lower. Similar patterns in the prevalence of teenage pregnancy and teenage motherhood were also observed. Compounded with high migration rates from rural areas to slums, the high fertility rate in slums would play a remarkable role in the growth of slums in developing countries (32). Hence, slum upgrading programs need to consider improving access to family planning services for women living in slum areas.

Awareness about HIV/AIDS was high in all urban settings, although comprehensive HIV/AIDS knowledge was lower in slums as compared to urban average. Sexual violence against women and high-risk sexual behavior, the two main factors fueling the HIV epidemic in urban areas, were higher in slums than non-slum areas. Other studies have also found that slum dwellers are at a heightened risk of HIV. The vulnerability of women to HIV infection was also high, especially when they become sexually experienced at very young ages (33).

Evidence about the prevalence of chronic diseases indicated that the prevalence of many lifestyle associated chronic diseases was significantly higher than in rural areas, whereas the prevalence of chronic disease associated with nutritional deficiencies was the reverse. Above all, slums suffer from remarkably high burden of tuberculosis (TB), especially in Mumbai. Other studies have also reported that obesity is high in urban poor both because of high energy intake and short stature that resulted from chronic malnutrition during childhood (34). The high prevalence of TB in slums was also consistent with the findings from other studies (35).

Among the several reasons that explain the differences in health and health-related indicators between rural and slum populations is that rural populations may have access to land and are able to grow food for consumption as well as generating income. Slum residents may or may not have access to land via extended family/community networks. Thus, slum residents may face health challenges that could have otherwise been averted if they had access to land. However, the effect of access to land on health in the study settings needs further study.

There were some limitations associated with this comparative analysis. First, the contexts of slums and non-slums in four countries were different, and this was not accounted for in the analysis. Second, there were variations in time of studies in countries where different data sources were used. These may contribute to some differences in indicator values. Third, there were some variations in the measurement of the indicators, which may affect comparison between countries for some indicators. Finally, data were not complete for some of the indicators. Despite these limitations, this is the first study that attempted to compare health indicators across the three residential domains at national level in multiple countries at multiple points of time. Although the limitations wouldn’t prevent comparison of the indicators, the readers are advised to take them into account in the interpretation of the results.

## Conclusion and recommendations

Early childhood mortality was worse in slums than rural communities, and child malnutrition was also higher in slums. The prevalence of childhood illnesses was also higher in slums than rural areas, but treatment seeking for these illnesses was better in slums. Slums had higher coverage of maternal health services than rural communities. Slums also had a lower fertility and higher contraceptive coverage than rural communities. Slum–rural difference in infant mortality was wider in Bangladesh than in Kenya. Analysis of differences in AACs yielded a mixed pattern. If the current trends continue, the situation may get reversed and slums will have better health status than rural communities in the next 10–15 years. Overall, mortality and morbidity indicators were worse in slums than rural communities. However, access to care and health service coverage indicators were better in slums than in rural communities.

Accordingly, further studies are needed to explore why slums have higher morbidity and mortality despite better access to health care. In addition, studies are needed to identify other determinants of health in rural populations that explain the relative lower morbidity and mortality rates despite lower level of access to health care. Finally, future studies need to consider collecting and using longitudinal data to compare health indicators among these residential domains.

## Paper context

A few studies cast doubt on the assumption that urban slum residents have worse health status when compared to other urban populations, but better health status than their rural counterparts. However, a systematic comparative analysis is lacking. This multicountry and multipoint study used nationally representative data to compare health indicators across the three residential domains. We report that slum populations have better access to care but worse health outcomes as compared to rural populations.
